# Experimental Early Stimulation of Bone Tissue Neo-Formation for Critical Size Elimination Defects in the Maxillofacial Region

**DOI:** 10.3390/polym15214232

**Published:** 2023-10-26

**Authors:** Nadezhda Nicolaevna Patlataya, Igor Nicolaevich Bolshakov, Anatoliy Alexandrovich Levenets, Nadezhda Nicolaevna Medvedeva, Vladimir Alexeevich Khorzhevskii, Mariya Arturovna Cherkashina

**Affiliations:** 1 Institute of Medicine and Biology, Moscow State Regional University, Moscow 105005, Russia; nadya_barahtenko@mail.ru; 2 Department Operative Surgery and Topographic Anatomy, Voino-Yasenetsky Krasnoyarsk State Medical University, Krasnoyarsk 660022, Russia; 3 Department Surgical Dentistry and Maxillofacial Surgery, Voino-Yasenetsky Krasnoyarsk State Medical University, Krasnoyarsk 660022, Russia; aalevenets@mail.ru; 4 Department of Human Anatomy, Voino-Yasenetsky Krasnoyarsk State Medical University, Krasnoyarsk 660022, Russia; medvenad@mail.ru; 5 Department Pathological Anatomy, Voino-Yasenetsky Krasnoyarsk State Medical University, Krasnoyarsk 660022, Russia; vladpatholog@yandex.ru; 6 Krasnoyarsk Regional Pathological and Anatomical Bureau, Krasnoyarsk 660022, Russia; 7 Pediatric Faculty, Voino-Yasenetsky Krasnoyarsk State Medical University, Krasnoyarsk 660022, Russia; mashacherkasha@mail.ru

**Keywords:** experimental critical size bone defect, maxillofacial area, chitosan, bone formation, morphometry, rats

## Abstract

A biomaterial is proposed for closing extensive bone defects in the maxillofacial region. The composition of the biomaterial includes high-molecular chitosan, chondroitin sulfate, hyaluronate, heparin, alginate, and inorganic nanostructured hydroxyapatite. The purpose of this study is to demonstrate morphological and histological early signs of reconstruction of a bone cavity of critical size. The studies were carried out on 84 white female rats weighing 200–250 g. The study group consisted of 84 animals in total, 40 in the experimental group and 44 in the control group. In all animals, three-walled bone defects measuring 0.5 × 0.4 × 0.5 cm^3^ were applied subperiosteally in the region of the angle of the lower jaw and filled in the experimental group using lyophilized gel mass of chitosan–alginate–hydroxyapatite (CH–SA–HA). In control animals, the bone cavities were filled with their own blood clots after bone trepanation and bleeding. The periods for monitoring bone regeneration were 3, 5, and 7 days and 2, 3, 4, 6, 8, and 10 weeks. The control of bone regeneration was carried out using multiple morphological and histological analyses. Results showed that the following process is an obligatory process and is accompanied by the binding and release of angiogenic implantation: the chitosan construct actively replaced early-stage defects with the formation of full-fledged new bone tissue compared to the control group. By the 7th day, morphological analysis showed that the formation of spongy bone tissue could be seen. After 2 weeks, there was a pronounced increase in bone volume (*p* < 0.01), and at 6 weeks after surgical intervention, the closure of the defect was 70–80%; after 8 weeks, it was 100% without violation of bone morphology with a high degree of mineralization. Thus, the use of modified chitosan after filling eliminates bone defects of critical size in the maxillofacial region, revealing early signs of bone regeneration, and serves as a promising material in reconstructive dentistry.

## 1. Introduction

The successive stages of bone regeneration are often a long-term, chronic process. It is known that with a large area of bone damage, physiological repair is not able to eliminate the defect on its own, and the use of many replacement polymer structures does not provide the expected regeneration effect [1,2,3]. The creation of functional bone using highly biocompatible substrates capable of self-organizing molecular architecture makes it possible to close large bone defects [4,5]. The starting material can be chitosan with a high degree of purification and deacetylation with a glomerular conformation of the molecule in reaction with chondroitin sulfate, hyaluronate, heparin, and alginate [6].

The idea of using the proposed polysaccharide construct is to form newly formed bone with signs of early bone formation [7]. Histological and immunohistochemical studies show that chitosan matrices create conditions for neovascularization in vivo at the early stages of implantation and are suitable for tissue engineering [8] in cases of early bone tissue regeneration when eliminating defects of a critical size, control of angiogenesis growth factors [9,10], and the early formation of highly vascularized periodontium [11]. This indicates the coupling of the processes of osteogenesis and angiogenesis [12]. Additional protonation of the amino group (NH_2_) in the chitosan molecule, for example, with weak ascorbic acid, makes it possible to increase the efficiency of controlling angiogenic reactions in the periodontium [13]. Physical cross-linking of biopolymers to obtain soft hydrogels with a three-dimensional structure allows for their use in tissue engineering [14] as delivery systems for hyaluronic acid and chondroitin sulfate, starch, and cellulose or their chemical derivatives to the area of damaged maternal bone tissue [15,16]. During long-term bone regeneration in the maxillofacial area, it is important to control the preservation of the structural integrity and function of the hydrogel matrix until new bone tissue is formed. It is very important that early active osteogenesis occurs under conditions of an anti-inflammatory response since the structure of chitosan forms electrostatic and concentration gradients for cells, metabolites, and water, which causes them to move toward the polymer. This reduces the degree of inflammation both at the site of polymer dislocation and in the peripheral zone [17]. An artificial structure based on natural polysaccharides and apatites can successfully solve this problem of regeneration of large bone defects with careful selection of a combination of polymers.

The authors of the article used the CH–SA–HA construct in this study, including a complex of polycationic and polyanionic polysaccharides: chitosan ascorbate; chondroitin sulfate; hyaluronate; heparin; serum growth factor (SGF); alginate; and hydroxyapatite. Each ingredient has its own reason to use to activate osteogenesis in the maxillofacial area. Physical or chemical synthesis of chitosan with other polymers significantly increases the mechanical strength and imparts elasticity to the hydrogel [18]. The mutual penetration of individual polymers forms a hydrogel network that strengthens the structure. Such combined meshes have been prepared using alginate and chitosan [19] or hyaluronic acid [20,21] for the purpose of bone tissue bioengineering. The combination of chitosan with an alginate–hydroxyapatite framework [22,23] stabilizes the gel matrix through the formation of polyelectrolyte complexes, binds the framework, and forms a compact structure. Earlier studies [24] demonstrated the role of SGF when added to a collagen–chitosan complex that contained most of the ingredients of the CH–SA–HA formulation (chitosan ascorbate, chondroitin sulfate, sodium hyaluronate, heparin sulfate). The presence of SGF in mouse embryonic fibroblast culture significantly increased DNA synthesis in the cells. This finding allowed the authors of this work to include this growth factor in the CH–SA–HA construct. It is noteworthy that delivery of growth factor to the area of bone regeneration requires preliminary protection from enzymatic hydrolysis of proteins. Such protection can be created using hydrogels and polysaccharide–protein scaffolds [25]. When growth factors and chitosan–sodium alginate copolymers are conjugated, prolonged kinetics of release into the medium is formed. A significant increase in growth factor sorption and release time is achieved by including a sulfated polysaccharide molecule, such as heparin, in the polymer. Different concentrations of heparin can regulate the rate of release of the active substance. The mechanism of prolongation is to prevent the interaction of anionic groups of alginate and growth factors. The inclusion of other polymers or inorganic compounds in the alginate composition is associated with a significant improvement in the mechanical and functional properties of the new structure. Favorable conditions for the regeneration of bone defects are observed with the implantation of an octacalcium phosphate–alginate construct [26]. At the same time, alginate selectively binds divalent cations, such as calcium and magnesium, which is the basis for the formation of a stable hydrogel [27]. It has been established that the inclusion of hydroxyapatite in scaffolds based on sodium alginate increases the stability of the scaffolds, provides access to the cell surface [28], improves biocompatibility, changes the microrelief of the matrix surface, and increases adhesion and migration of osteoblasts [29]. Scaffolds based on alginate and hydroxyapatite enhance the local healing of bone defects since they do not cause an inflammatory effect [30]. Such constructs ensure good attachment and proliferation of preosteoblastic cells and differentiation of osteoblasts [31]. The inclusion of the apatite component in the structure of chitosan is supposedly intended to stimulate the vascular endothelium during the exchange of calcium and phosphate already at the early stage of angiogenesis [32]. Due to the high chemical solubility of apatite structures with the release of ionized calcium and phosphates into the environment, this leads to the direct induction of angiogenesis [33] and the formation of vascular branches [34]. Thus, researchers associate the direct induction of angiogenesis with the participation of the calcium–phosphate environment with the direct activation of fibroblasts capable of secreting angiogenic growth factors (VEGF, βFGF, and TGFβ). This result creates favorable conditions for the adhesion, translation, and proliferation of endothelial cells of the great vessels with the preservation of their vascular phenotype [35]. Temporary stability of the polysaccharide matrix in tissue fluid is important for the full launch of the process of osteogenesis. Thus, the purpose of this study was to obtain morphometric evidence of early bone tissue regeneration in comparison with classical control in a bone defect of critical size. The novelty of this experiment lies in the creation of a new design that can be introduced into wide dental (and not only) practice in order to eliminate bone defects of critical sizes of various origins in patients. The hypothesis of effective early osteogenesis is to obtain the effect of early vascular endothelialization not only on the walls of the bone defect in the presence of a polysaccharide matrix but also on the endothelialization of the matrix itself. The selection of polysaccharides with a certain molecular weight and dose, the synthesis method to control the rate of degradation, as well as obtaining the effect of rapid formation of the spongy and compact part of the bone pose the immediate challenges of regeneration.

## 2. Materials and Methods

All manipulations with the animals were performed following the regulations specified in the Guide for the Care and Use of Laboratory Animals (National Research Council, 2011). The work was approved via complex scientific theme No. 01201362513 (2013/01/01–2021/01/01) “*Fundamental and applied scientific and technical developments of nano-level biopolymer structures and technologies for their production for use in cell and tissue engineering in socially significant human diseases*”; Section “*Dentistry*”. Research topic: “Restoration of the structure of the bone tissue of the maxillofacial region using polysaccharide polymers with extensive traumatic defects in conditions of sub-compensated diabetes mellitus.” Bioethical Commission for Working with Animals by the Ethics Committee of the Voino-Yasenetsky Krasnoyarsk State Medical University of the Ministry of Health of the Russian Federation (Protocol No2 of 10/28/2019).

### 2.1. Composition of CH–SA–HA Construction

The Developer is SBEU HPT Krasnoyarsk State Medical University, Russia. Gel mass of chitosan–alginate–hydroxyapatite (CH–SA–HA) containing a 2% solution of chitosan ascorbate (CH) (dissolution of the polymer in ascorbic solution acid in a ratio of 1:1.5) with a molecular weight of 695 kDa and a degree of deacetylation of 95% (a triply purified chitosan obtained in Vostok-Bor-1, Dalnegorsk, Russia. Specifications (No 9289-067-004721224-97), including per 1 g of dry chitosan ascorbate, 5–100 mg of sodium chondroitin sulfate (Sigma, St. Louis, MO, USA), 10–100 mg of sodium hyaluronate (Sigma), 2.5–5 mg of heparin sulfate (Russia, Pharm. Art. (No 42-1327-99), 110 mcg/d serum growth factors in cattle “adgelon” (SLL “Endo-Pharm-A”, Moscow region, Schcholkovo, Russia, Specifications (No 113910-001-01897475-97)), 4% sodium alginate (Pharm. Art. No 42-3383-97 or Specifications (No 15-544-83; Arkhangelsk algal plant. Co., Arkhangelsk, Russia)), including 50 wt% amorphous hydroxyapatite (5–20 nm, Russia, Pharm.Art. No 42-3790-99 or GOST 12.1.007-76) (SA-HA), in the ratio of chitosan ascorbate and sodium alginate 1:1 (CH–SA–HA) [36]. The gel mass was placed into vials in a volume of 2 mL, lyophilized, and sterilized electronically. 

An amount of 7.2 g of chitosan was added to the prepared solution of ascorbic acid (Pharm. Art. No 42-2668-95) with stirring at a temperature of +20–22 °C; the mass was stirred for 4–5 h until the chitosan was completely dissolved. Aqueous solutions of sodium salts of chondroitin sulfuric acid, hyaluronic acid, and heparin were successively added to the resulting 4% chitosan solution with constant slow stirring using a magnetic stirrer in a total volume equal to the volume of chitosan ascorbate. The introduction of each subsequent ingredient was carried out after the homogeneous mixing of the previous one with the chitosan gel. As a result, a 2% chitosan poly-ionic complex was obtained. Next, a 4% aqueous solution (gel) of sodium alginate was prepared, and 50% (by dry weight) of hydroxyapatite was added. The finished chitosan solution was thoroughly mixed with the sodium alginate solution using a high-speed mixer.

### 2.2. Experimental Animals

The conditions of biological test systems in the CDI CI correspond to *Guide for the Care and Use of Laboratory Animals*, 8th edition, 2011, NRC, USA (*Manual on the content and use of laboratory animals*, 8th edition, 2011, National Research Committee, USA). The maintenance of animals was in individually ventilated cells from poly-sulfone Sealsafe, 461 × 274 × 228 mm (production TECHNIPLAST. P. A.). The rooms contained biological test systems, controlled temperature (+18–24 °C), humidity (30–70%), illumination (12/12 h), and the multiplicity of air (XII without recirculation). Control of climatic parameters was carried out in accordance with the SOP “control of climatic parameters in the premises of the vivarium”. The distribution of feed and water was carried out at a fixed time; the change of litter was made once a week in accordance with the SOP “preparation of cells for biological test systems. Marking. Change of bedding, feed, water”.

### 2.3. Modeling Defects of the Critical Size of the Mandibular Angle in Rats

This study was conducted on 84 female Wistar rats weighing 200–250 g. Laboratory animals were divided into 1 control and 1 study group. Each group was subjected to a morphometric study at 3 and 5 days and 1, 2, 3, 4, 6, 8, and 10 weeks. The study group included 40 animals, in which an extensive defect in the lower jaw was modeled, followed by the filling of the bone cavity with a polymeric construct: chitosan ascorbate–sodium alginate–hydroxyapatite (CH–SA–HA). The control group included 44 animals with an identical bone cavity filled with a natural intraoperative blood clot at the same time of observation. Anesthesia was performed using a mixture of tiletamine/zolazepam at a dose of 2 mg/rat (Zoletil 100, Vibrac, France) and xylazil (Rometar, Bioveta, Czech Republic) at a dose of 0.02 mL/rat in a ratio of 1:1. The mixture was injected in a volume of 0.5 mL intramuscularly, then the skin in the lower jaw area was treated using an aqueous solution of chlorhexidine, an incision of 1.5–2 cm was made on the lower jaw in the area of the masticatory ridge along masseter and periosteum; a rounded three-wall defect 4 × 5 × 5 mm^3^ in size was applied on the masticatory ridge with a dental spherical burr. While burring, the bone was cooled with an aqueous 0.2% chlorhexidine solution (series CHG-013/17 J. Amphrey Laboratories, Maharashtra, India). The cavity of the bone defect was dried with tampons and filled with CH–SA–HA lyophilic mass or auto-blood clot formed when bleeding from a wound; the defect was closed with periosteum, and the skin was sutured with separate 5.0 monofilament sutures. The wound was treated with 1% aqueous solution of chlorhexidine. In order to prevent the development of the inflammatory process, all animals were injected intramuscularly with the antibacterial drug ceftriaxone at a dose of 8 mg/rat and the antihistamine drug Suprastin at a dose of 0.1 mg/rat. Within three days after surgery, the animals received Tramadol 2.5 mg 2 times a day. During the first day, access to water was provided. Feeding was carried out 24 h after the operation with an exclusively liquid mixture, “Polyproten-Nefro”, using soy protein Supra 760 (USA) (LLC “Protenfarma”, Moscow, Russia) for 3 days. Medical support was provided by a broad-spectrum antibiotic (ceftriaxone at a dose of 8 mg/rat), antispasmodics, and vasodilators. The sutures were removed on the 7th day after surgery.

### 2.4. Morphological Analysis of Bone Tissue

The mandibles of the animals were placed in a 10% solution of buffered formalin; then, fragments of the bodies of the mandibles were separated in the area of the postoperative defect with the capture of unchanged bone along the perimeter for 5 mm and placed in the decalcifying solution “Trilon B” for a period of 24–48 h. The decalcified fragments were dehydrated in increasing concentrations of isopropyl alcohol and embedded in paraffin wax. On a MicroTec CUT4050 microtome, serial sections (20–25 sections) of 4–5 µm in thickness were made in the transverse plane (relative to the animal’s body) through the entire area of the bone defect in the area of its outer and inner walls. Sections were stained with hematoxylin–eosin and picrofuchsin, according to van Gieson. The morphological study was carried out on an Olympus BX45 microscope with an Olympus DP 25 attachment for photo–video documentation and the Cell^D software package, as well as with scanning histological preparations in a FLASH 250 3D HISTECH histoscanner (Hungary). The histo-morphometric evaluation was performed on digital micrographs, which were obtained using the software “Cell^D” and “NIS-Elements Document”, while morphometric measurements were performed in the program “JMicroVision 1.2.7”. Digital histological sections obtained as a result of scanning micro-preparations in a histoscanner were evaluated using the CaseViewer Ver.2.3 Build 2.3.9.99276 3D HISTECH software (Hungary).

Bone tissue recovery was assessed according to the following histomorphometric criteria: volumetric density of bone and connective tissue (BV (%), the percentage ratio of the volume occupied by bone structures to the total volume of the histological section); thickness of bone trabeculae (BTT) (mm), the criterion stipulating that the bone trabecula is a thin plate, with measurements taken between the edges of the bone trabecula (5–8 measurements in relation to each trabecula with the calculation of the median); inter-trabecular spaces (ITS) (mm), the distance between the edges of the cancellous bone trabeculae, the calculation was made in accordance with the so-called parallel plate model—(BV) minus (BTT) (mm); volumetric density of osteoid and vessels (OS) (%), the percentage ratio of the surface of bone trabeculae occupied by osteoid to the total bone surface); numerical density of inflammatory cell infiltrate (%) (fibroblasts, segmented leukocytes, histiocytes, and giant multinucleated cells); bulk density of the implant (%); free bone surface area (FS) (%), the percentage of the non-eroded surface of bone trabeculae and the surface not occupied by osteoblasts, osteoclasts to the total bone surface); area filled with osteoblastic cells of bone trabeculae (OBS), the percentage ratio of the surface of bone trabeculae occupied by osteoblasts to the total bone surface); eroded (osteoclastic) surface of bone trabeculae area (ES) (%), the percentage ratio of the surface of bone trabeculae with the formation of gaps to the total bone surface, including the surface occupied by osteoclasts. The numerical density of cell structures was measured as the number of cells in relation to the area of the field of view. The relative density of the measured structures was determined by the following formula: RDS (%) = (Sa/St) × 100, where Sa is the total area of all selected areas, and St is the area of the digital image.

### 2.5. Statistical Analysis

**Figure 1:**
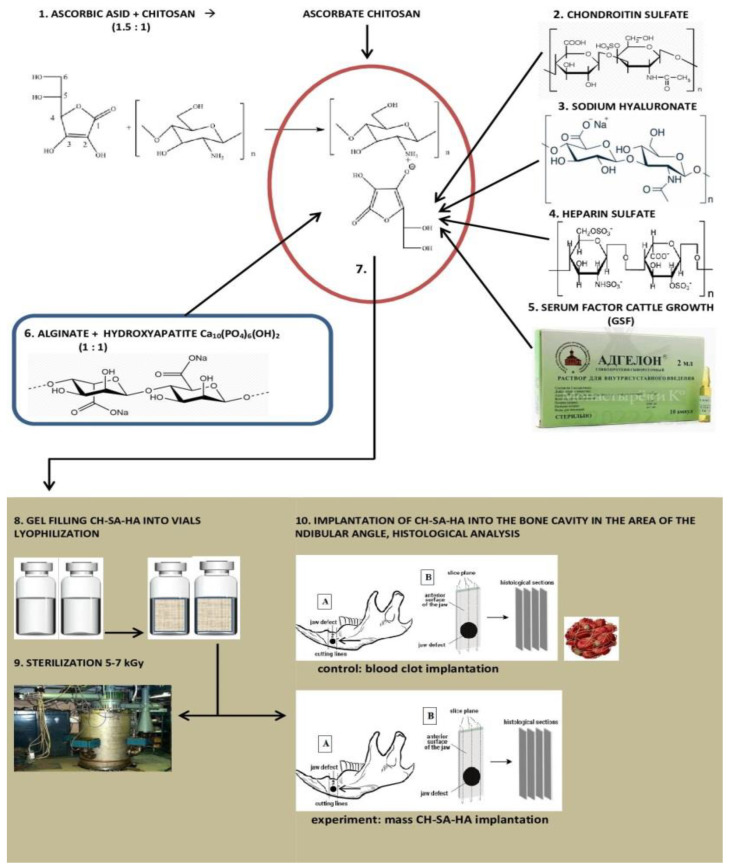
Experimental study design.

Statistical analysis of data and the creation of graphic illustrations were carried out using the free software computing environment “R, version 4.2.1” and the programming language “R”. The assessment of the obtained variables in relation to compliance with the normal (Gaussian) distribution was carried out on the basis of the Shapiro–Wilk test, as well as on the basis of the graphical method (Quantile–Quantile plot). Most of the variables obtained obeyed the normal distribution law, and the cases of deviation of the variables from the normal distribution were not pronounced. For variables deviating from the normal distribution, the following methods of data transformation were used to achieve compliance with the normal distribution: with right-sided (positive) skewness, square roots were taken from the obtained values, and in the case of left-sided (negative) skewness, the formula $\sqrt{max\left(x+1\right)-x}$ was used, where “x” is the value obtained. Descriptive statistics of the obtained data were presented as median, 25%, and 75% quartiles (Me[Q_1_;Q_3_]). During the choosing statistical tests for assessing the type I error (α) and the sensitivity of the criterion (1 − β), the following parametric methods of statistical analysis were used: one-way analysis of variance (ANOVA) for paired comparisons of independent variables; Welch’s *t*-test was used for multiple comparisons; we used Bonferroni amendment. The presented variants of assessments were carried out taking into account the equality of variances, the variables under study, as well as taking into account the level of sensitivity of the criteria not lower than *p* = 0.75. To assess the error of the first kind, taking into account the small volume of the studied samples, the threshold value p = 0.01 was used. The design of the pilot study is shown in Figure 1.

## 3. Results

### 3.1. Morphological Analysis of Bone Tissue Restoration in the Early Stages in the Defect Zone

**Figure 2:**
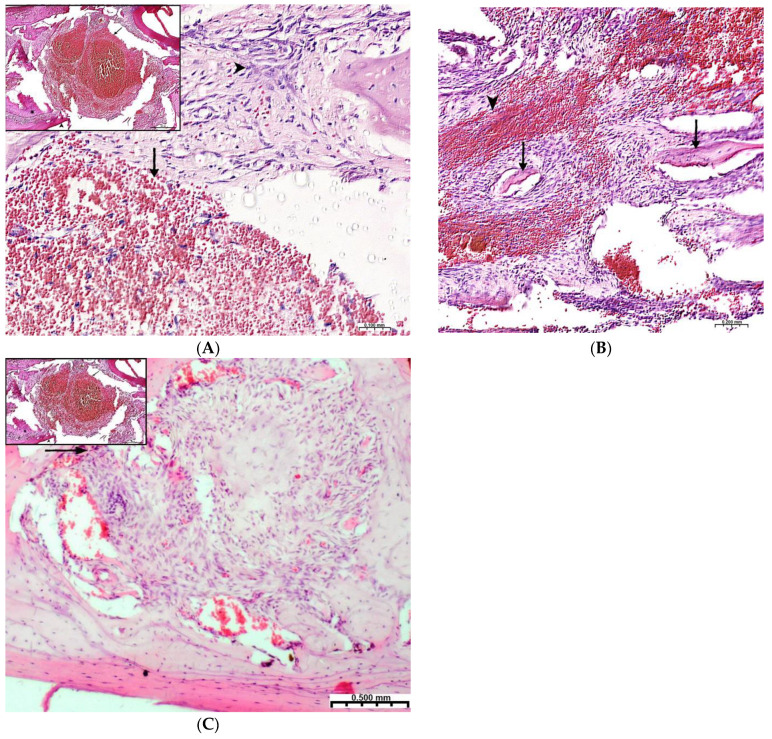
Regeneration of a bone defect in the lower jaw at the early stages of this experiment. (**A**)—experimental group; day 3 of this experiment, there is no bone tissue at the site of the defect; mesenchymal stromal cells (**^**) migrate to the area of the blood clot (**↑**) for further differentiation into osteoblasts; in the upper left corner of the control group, day 3 of this experiment, a formed defect with a blood clot (↑) is visible; the periodontal ligament (*) and the cortical plate of the lower jaw (**^**) are visible; (**B**)—CH–SA–HA group, day 7 of this experiment, islets of regenerating bone tissue (**↑**) in the area of hemorrhagic impregnation (**^**); (**C**)—control group, day 7 of this experiment, the bone defect is filled with immature connective tissue (**↑**).

Plain microscopy of histological preparations in the area of the postoperative defect of the lower jaws of healthy rats removed after 3 days of this experiment revealed extensive foci of hemorrhagic impregnation (Figure 2A, upper left corner) in combination with severe inflammatory infiltration and a clear predominance of segmented leukocytes. Among other cells of the inflammatory infiltrate, the presence of lymphocytes, histiocytes, fibroblasts, and plasma cells was noted. There were also visualized signs of the formation of granulation tissue with a pronounced edematous extracellular matrix and numerous thin-walled capillaries. In histological preparations from the group with implanted “CH–SA–HA” biopolymer, along with the changes described above, abundant, diffusely distributed amphophilic granules of a foreign substance were determined. In histological preparations on the 5th and 7th days of this experiment, a similar microscopic picture was noted with an important feature, which was characterized by the appearance of eosinophilic “islands” of bone tissue formation with the presence of cubic and polygonal cells on the surfaces of cells with weakly basophilic cytoplasm and spherical nuclei–osteoblasts (Figure 2B). Single large cells with multiple spherical nuclei and homogeneous “foamy” cytoplasm–osteoclasts were also detected. In some preparations, these cells were absent. In separate preparations on the 7th day of this experiment of both study groups, along with bone tissue, the formation of small areas of cartilage tissue was noted. In the walls of the bone defect, the migration of mesenchymal stromal cells toward the blood clot is typical in the earliest stages (Figure 2A). For the control group, after 1 week, only the filling of the bone cavity with immature connective tissue is typical (Figure 2C). Thus, the processes of bone formation, even in the early stages, are the most active in the experimental group of animals.

The assessment of the numerical density of the inflammatory infiltrate was made per unit area—0.043 mm^2^, which corresponded to the area of the field of view displayed on the monitor screen at × 400 magnification. The presented histomorphometric criterion was not studied after the experiment period of 3 weeks due to the complete elimination of pathomorphological signs of inflammation. As shown in Table 1, in multiple comparisons using one-way analysis of variance, there was a significant influence of factors such as the timing of this experiment and group affiliation at each time interval of this experiment. Indicators of the numerical density of the inflammatory infiltrate in the early stages after surgery significantly increased daily in both studied groups (*p* < 0.01), while in the group of animals with implanted biopolymer “CH–SA–HA”, the objective indicator of cell infiltration from the 3rd to the 7th day of this experiment increased by 57% (*p* < 0.01), while in the control group, the same range of changes was only 36% (*p* < 0.01) (Table 1). 

Thus, the processes occurring at week 1 correspond to the first stage of bone tissue regeneration (the formation of an osteoid island) and are characterized by the appearance of inflammatory infiltrate cells, the number of which increases every day, and osteoblastic differon cells that trigger the process of osteogenesis. When comparing the ongoing process of regeneration in the animals of the control and experimental groups, it follows that in the animals of the experimental group, the inflammation process is less pronounced; osteoblasts appear in greater numbers earlier, and osteoid islands are formed as early as in the first week.

### 3.2. Regeneration of Bone Tissue in the Defect Area within 2–4 Weeks

After 2 weeks of observation in both study groups, there was a more than twofold decrease in the volume of the numerical density of the inflammatory infiltrate (*p* < 0.01). In pairwise comparisons, the numerical density of the inflammatory infiltrate in the experimental group was significantly lower (*p* < 0.01) at all estimated time intervals of the experiment, except for the period of 3 weeks. In pairwise comparisons, the digital values of the numerical density of the inflammatory infiltrate in the peripheral zone of the bone defect (experiment) were more than two times lower (*p* < 0.01) compared with the values in the zone of the bone defect proper (Table 2).

The application of a large bone injury in the lower jaw triggers the mechanism of early bone resorption in both groups with an active osteoclastic reaction, especially at the periphery of the defect (Table 1, Table 2 and Table 3), followed by active replacement with new bone. The activity of bone resorption in the peripheral zone exceeds 12–15 times when compared with the area of the bone defect. The control group of animals is characterized by a long reaction of bone destruction with a slower formation of spongy and especially compact bone (*p* < 0.01). 

**Figure 3:**
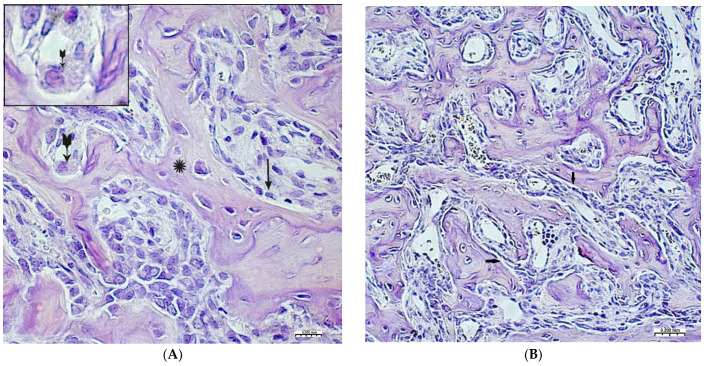
Changes in the area of the bone defect characterizing the active phase of bone tissue regeneration in healthy and experimental groups of rats: (**A**)—CH–SA–HA group, 2nd week of the experiment. On the surface of the bone beams (*), multiple osteoblasts are visible (**↑**), and a formed gap with an osteoclast is also visible (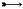). Hematoxylin–eosin staining; (**B**)—control group, 2nd week of the experiment; the osteoblastic surface of the bone beams is significantly smaller in comparison with the experimental group (**↑**). Hematoxylin–eosin staining.

On the 2nd and 3rd weeks of the experiment, formed bone beams were visualized in the area of the inflicted defect, on the surfaces of which numerous osteoblasts were detected, mostly of a cubic shape (Figure 3A,B). The volume density of bone tissue and the osteoblastic surface during these periods prevail in the preparations of the experimental group animals (Table 2). The volume of surfaces of bone beams occupied by osteoblasts in preparations of 3 weeks was noticeably lower when compared with preparations of previous time intervals. In both study groups, few osteoclasts were detected with the formation of characteristic lacunae (Figure 3A). The structure of most bone beams was characterized by ordered fibers. The density of cellular infiltrate, in comparison with the previous time intervals, was noticeably lower among the dense connective tissue structures and was characterized by mild infiltration, mainly by histiocytes and fibroblasts. In the group of animals after CH–SA–HA implantation, diffusely distributed, finely dispersed amphophilic granules containing a polysaccharide construct were detected. 

**Figure 4:**
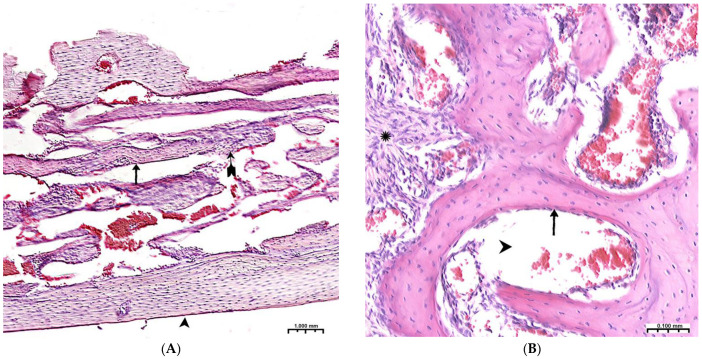
(**A**)—The control group, 3rd week of the experiment, formed thin bone trabeculae in the area of the defect (**↑**) with pronounced intertrabecular spaces (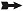); bone trabeculae are covered with cell mass (**^**); (**B**)—CH–SA–HA group, 4 weeks. Visible bone beams with predominantly free surfaces (↑) and intertrabecular spaces (**^**); the adjacent peripheral area with the growth of maturing connective tissue (*). Hematoxylin–eosin staining.

By the end of 1 week after surgery, the volumetric density of bone tissue increases many times, both in this experiment and in the control. However, starting from the 2nd week, the value of BS in this experiment significantly exceeds the control results. In fact, in the first month of the experiment, the bulk of the newly formed bone tissue with high growth rates of the spongy and compact components is laid in this experiment (Table 2). A high inflammatory reaction in the intrinsic zone of the bone defect is combined with a low level of inflammation in the peripheral zone and a stable picture of bone tissue volume at a distance of 5 mm from the zone of inflammation in the first 6 weeks of this experiment (*p <* 0.01). Filling the surface of the bony trabeculae with cell mass leads to the thickening of the trabeculae and contraction of the intertrabecular spaces and the inactive free bone surface. In the first 2 weeks, the increase in the thickness of bone trabeculae in this experiment was 100%, and in the control, 83%. The walls of the bone cavity in control after 3 weeks are in the form of thin bone beams with pronounced intertrabecular spaces and poor cell mass (Figure 4A). 

The 4th week in the experimental group shows a newly formed bone in the defect zone; a large proportion of the surfaces of the bone beams are already free from cells, which indicates a partial process of completion of osteogenesis. (Figure 4B). However, in almost all periods of registration, the spongy part of the bone wall of the defect in the experimental group was filled significantly higher with osteoblastic cell mass (Table 1, Table 2 and Table 3). The analysis shows that after 4 weeks, a late final stage of bone resorption is formed, as indicated by a significantly higher number of osteoclastic-type cells with the formation of lacunar surfaces. In the bones of the experimental group of animals, signs of small focal accumulations of the implanted polymer are preserved.

Thus, the follow-up period of 2–4 weeks corresponds to the following stages of osteogenesis (osteoid and osteoid calcification stages), during which spongy bone is formed, represented by bone trabeculae and wide inter-trabecular spaces filled with developing bone marrow. Reticular fibrous bone tissue is involved in the construction of this bone. The processes occurring during these periods are characterized by a decrease in the number of inflammatory infiltrates and high osteoblastic activity. Animals of the experimental group in the indicated time periods are characterized by a high volumetric density of the newly formed bone tissue.

### 3.3. Regeneration of Bone Tissue in the Defect Area within 6–10 Weeks

**Figure 5:**
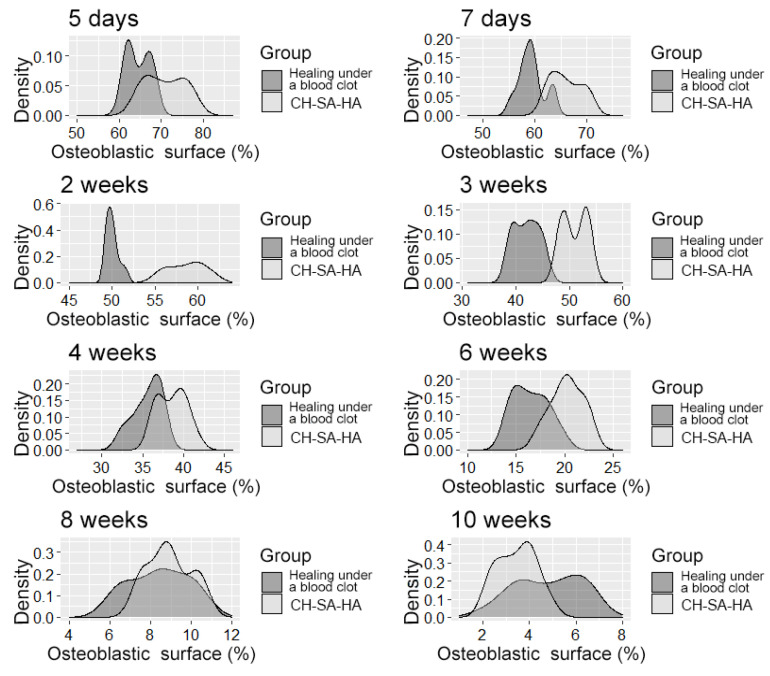
The density distribution of the variable is “osteoblast surface”.

**Figure 6:**
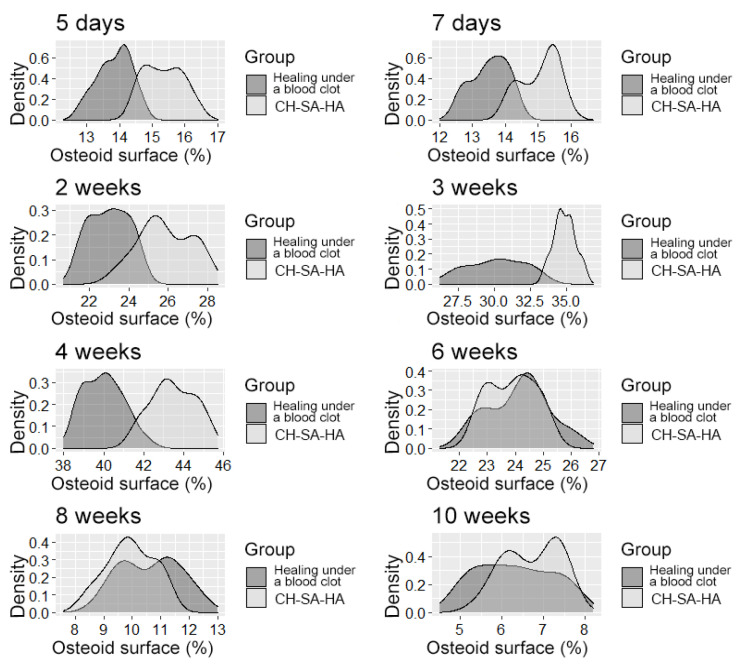
The distribution density of the variable is “osteoid surface”.

After 6 weeks (Table 3), the activity of bony malacia processes sharply and steadily decreases to its original value. Active early and prolonged (within 6 weeks) osteoblastic reaction in the experimental group provides early formation of the osteoid matrix, ready for the mineralization of new bone. The processes of osteoblastic reaction and osteoid formation proceed dynamically and synchronously (Figure 5 and Figure 6).

**Figure 7:**
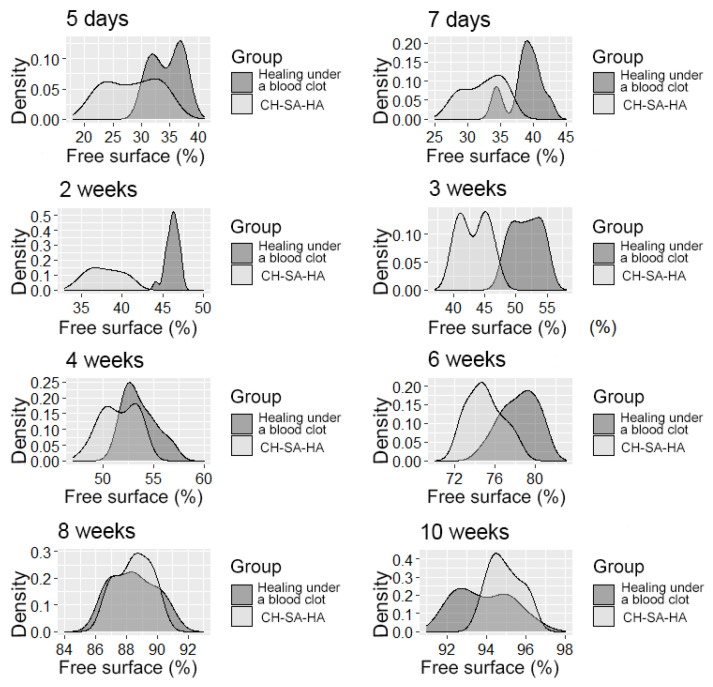
The distribution density of the variable is the “free surface” in the studied groups at different times of the experiment.

It should be pointed out that the activity in the dynamics of osteogenesis in the control group is significantly lower than the results of this experiment. The inactive zone of the bone cavity walls (FS%) occupies a significant area during the first 6 weeks of the regeneration process (Figure 7).

**Figure 8:**
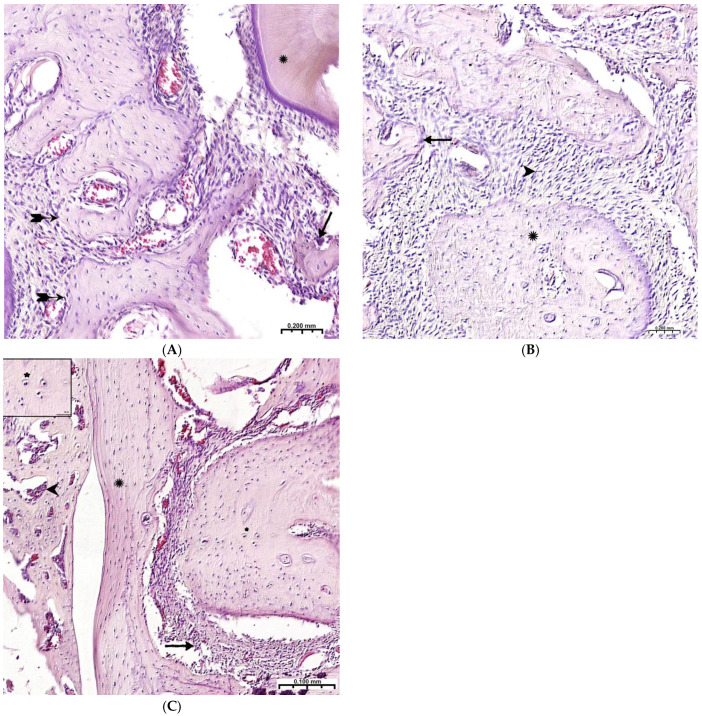
Late terms of reorganization of the bone cavity in the experimental group. (**A**)—6 weeks, oriented bone beams with a mass of flattened osteoblasts (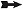), adjacent peripheral area with proliferation of maturing connective tissue, active vascular endothelialization of bone regeneration, single osteoclasts (**↑**), organization of compact bone and periodontal ligament (*); (**B**)—8 weeks, large amount of spongy (**^**) and compact (*) bone tissue with cellular reaction on the bone beams (**↑**) and the formation of many micro-vessels; (**C**)—experimental group, 10 weeks, the mandibular defect is closed by a compact bone, in the border zone there is mature connective tissue (**↑**), in the adjacent bone tissue (*)—reactive bone marrow structures (**^**), formed osteons (*), many Haversian canals with a vascular component; Hematoxylin–eosin staining.

During the 6th and 8th weeks, spongy bone tissue predominates at the defect site with oriented bone trabeculae, flattened osteoblasts, and single osteoclasts (Figure 8A). Later, 6 weeks after implantation of the CH–SA–HA biopolymer, rare small-focal accumulations of amphophilic granules with graft remnants were determined. After 8 and10 weeks, foreign bodies were not detected. An important result is the active closure of a bone cavity of a critical size with full-fledged cancellous and compact bone, with formed osteons and Haversian canals with a vascular component (Figure 8B,C).

Thus, 6–10 weeks of this experiment correspond to the last stage of osteogenesis (formation of a compact bone or differentiation of reticufibrous bone tissue into lamellar bone tissue). At 6–8 weeks, high osteoblastic activity is still observed; the volume of the resulting bone increases, and osteoclast cells are active, participating in the processes of tissue reorganization. All of these processes are most active in the experimental group of animals. By the 10th week of the experiment, lamellar bone tissue is formed, which is characterized by the presence of osteons in the bone trabeculae and the formation of Haversian canals. Animals of the experimental group are characterized by a high volumetric density of the emerging bone in earlier periods of regeneration.

**Figure 9:**
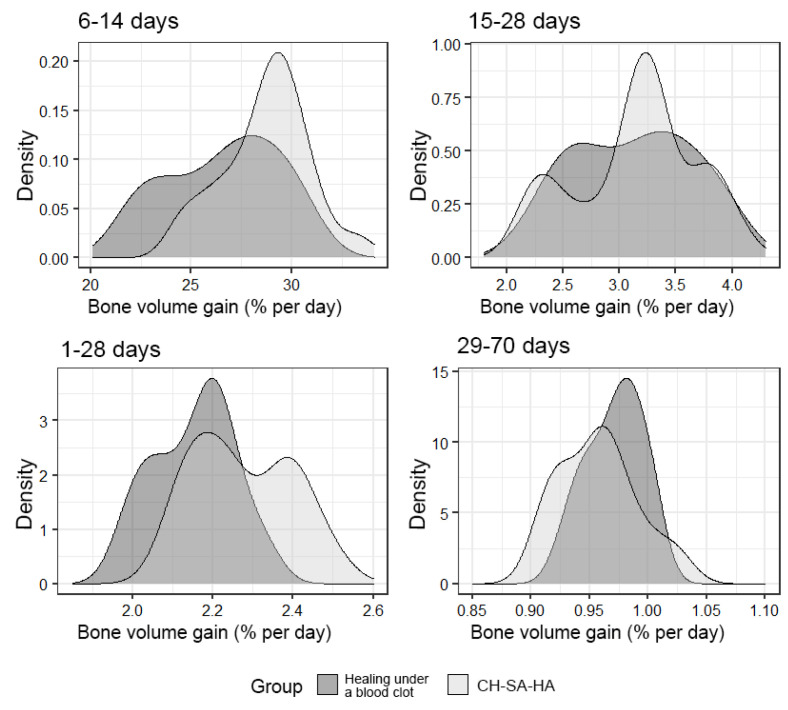
The density of the distribution of the variable, “increase in bone volume”, in the study groups at randomly selected intervals of the experiment.

An important final result of bone defect regeneration is the rate of bone tissue growth as a percentage for 1 day. The results show that with an arbitrary choice of time intervals of 1–28 days, 6–14 days, 15–28 days, 29–70 days, 1–70 days, the activity of osteogenesis processes changes significantly. One-way statistical analysis (ANOVA) established the significance (*p* < 0.01) of the influence of the experimental period factor on the variable under consideration. Even though the growth rate for the entire period of this experiment (70 days) was 0.94% per day in the group with implanted CH–SA–HA biopolymer and 0.96% per day in the control group, a high rate of bone tissue growth in the focus regeneration was observed in the time interval of 6–14 days. In the group with implanted biopolymer, the growth rate was 28.9% per day, and in the control group, this figure was 27.2% per day. Statistical analysis based on Welch’s *t*-test showed that this difference was significant (*p* < 0.01). Analysis over a randomly selected period of 1–28 days also revealed significant differences (*p* < 0.01) in favor of the group of animals implanted with CH–SA–HA biopolymer (Figure 9).

## 4. Summary

Early specialization of the cell mass on the walls of bone defects, proliferation, and transition of osteoblasts into the structure during its biodegradation with rapid rearrangement of the bar structure and the formation of spongy bone is a characteristic reaction to the implantation of a complex polysaccharide matrix. Such a preliminary result is very important when it comes to eliminating an extensive bone cavity in a significant reduction in inflammatory infiltration in the area of damage, given the specificity of the microbial flora in the oral cavity. It should be noted that during the period of 70 days of this experiment, the most active periods of osteogenesis were revealed, which occupy the earliest period of bone formation compared to the control. The encouraging results of this study form a promising direction not only in maxillofacial surgery but also in the field of dental transplantology, where tight contact between implants and bone is required.

Analysis of the results of regeneration of the spongy and compact parts of the bone in the works of other authors shows that the use of the product of the apatite base of the bone octacalcium phosphate (Ca_8_H_2_(PO_4_)_6_·5H_2_O), found in human dentin, is due to a higher activity of inclusion in the early process of bone regeneration [37]. However, during the implantation of such a construction into a human lower jaw, a defect of 40 mm long formed after the removal of a residual cyst with apical periodontitis, leading to the filling of the bone defect in the alveolar region within 12 months against the background of the formation of the vascular network [38].

The inclusion of chitosan in the complex, in addition to its other useful properties, is also due to the launch of the vascular network formation [39].

The non-inclusion of collagen in the CH–SA–HA construct in this work is due to the initially poor mechanical properties of the polymer, which limit its use in biomedicine [40,41]. Despite the excellent biodegradable and high adhesive properties of collagen, maintaining active cell growth [42], the mechanical properties of collagen matrices are difficult to tune in a wide range of values; collagen itself is not very well amenable to direct chemical modification without affecting its structure or biological activity. The triple helical structure of collagen is easily destroyed by environmental conditions (e.g., pH, temperature), and the number of engineering manipulations that can be achieved using collagen is limited [43].

However, when protein ingredients are included in the constructs, there is always a risk of intensifying the inflammatory reaction in the defect zone and, as a result, subsequent destruction of both the maternal and newly formed tissues. For example, the inclusion of fibrin as a matrix is designed to support the initial stages of tissue regeneration. Despite the presence of this substrate, which is naturally associated with vasculature growth factors, increased fibrosis, and scar tissue formation cannot be ruled out. This reaction prevents physiological tissue regeneration [44].

Nevertheless, the authors in their works do not refuse to use type I collagen in combination with polysaccharides. Intermolecular cross-linking using physical or chemical methods [45,46] improves the framework properties of collagen. Co-polymerization of collagen and chitosan successfully solves this problem [47], including the protective functions of macrophages and fibroblasts [48] and a significant increase in the biostability and antimicrobial activity of collagen.

Numerous publications on bone regeneration after the formation of a critical-size bone cavity show that self-implantation of a multilayer microfiber of chitosan with a molecular weight of 200–300 kDa and a degree of deacetylation of 90% into a 5 mm femoral defect using mesenchymal stem cells differentiated in osteoblasts within 12 weeks does not lead to the final filling of the defect. Compared with the use of a bone allograft, the results of the regeneration volume were 1.5 times lower (*p* < 0.01) [49]. A blinded assessment revealed a mean score of 4.4 ± 1.3 for the implants made of chitosan and 5.9 ± 0.8 for bone allograft. The results showed strong statistical significance (*p* < 0.0001), as determined by one-way ANOVA. At the same time, a comparative analysis of the formation of vessels in the regeneration process did not reveal the advantage of either chitosan or bone allograft (as determined by one-way ANOVA, *p* = 0.1419). A comparison of data from the experimental groups yielded no statistically significant differences in terms of osteoclast numbers (one-way ANOVA; *p* = 0.7474).

Counting the number of osteoblasts in the defect zone revealed a clear advantage of the bone allograft (one-way ANOVA; *p* = 0.0077). The difficulty of closing large bone defects is confirmed in detailed studies by Casillas-Santana, M.A. et al., 2023. Using colloidal chitosan in combination with nano-hydroxyapatite or silver nanoparticles in this study, the authors did not achieve complete regeneration of a bone defect of a critical size after 6–8 weeks of observation and achieved only 75% of healing [50]. This result was rated by the authors as very good. The low result of regeneration in many studies is probably due to the absence of anionic polysaccharides in the chitosan structure; for example, sodium alginate, chondroitin sulfate, hyaluronate, and heparin in certain weight ratios that modify chitosan into a nanocomponent of the complex. Prospects for the use of complex chitosan constructs lie in the ability of the polymer itself to induce active osteogenic differentiation of stem cells and retain this property not only in vitro but also in vivo. Such complex polymer structures with the inclusion of growth factors (for example, alginate + VGF_165_, BMP_2_) and a program of simultaneous elution into the bone cavity will allow for closing bone defects of critical size of any localization [51]. Current reviews on the problems of periodontal restoration indicate that further extensive research is required, with a need to focus on improving the biological interfacing between the graft material and the host tissues. Further approaches in the field of periodontal regeneration will rely on a combination of therapies using improved biomaterial options [52].

## 5. Discussion

Obtaining a morphological picture of early bone formation due to the active formation of the osteoid surface, starting from 5 to 7 days after surgery, and confirmation of this process during the first month after injury indicates the role of the polymer structure in the advanced regeneration of the cancellous part of the bone. The authors believe that the creation of specialized technologies and technical means for obtaining osteogenic matrices for direct transplantation into bone tissue defects in animals and humans is very important for subsequent widespread use in medical practice. Successfully constructed osteogenic matrices, when in contact with the wall of the bone cavity, are able to reduce or even exclude certain stages of osteogenesis, for example, the cartilaginous stage of development, and trigger the mechanisms of early bone formation. The absence of a well-perfused substrate in the affected area in the early stages after extensive trauma to the bone tissue, the lack of the ability of synthetic materials that do not contain growth factors and osteogenic cells to cover large bone defects of a critical size [53] is one of the main problems in the long-term restoration of the integrity of cancellous and compact bones. Preservation of the viability of the mass of cellular material over a large extent of the bone defect should consist of obtaining a highly vascularized module in an artificial matrix and creating conditions for its integration into hard tissue. The formation of micro-vessels is the basis for the physiologically active process of bone formation [54]. Despite the successful solution to the issue of the beginning of early bone formation, the first stage of the process of reconstruction of the vascular network remains insufficiently understood from the point of view of the mechanism of contact interaction between the vascular endothelium and artificial or natural polymers. If polymer matrices in the form of nanoparticles are used (this design was used in the work of the authors), then this enhances the efficiency of the delivery of vector molecules to cells, leads to overexpression of angiogenesis molecules, and enhances endothelial recruitment and new formation of blood micro-vessels [55]. Stimulation of proliferation and translation of the vascular endothelium occurs outside the vascular wall into the tissue compartment [56,57,58]. Using this postulate, it should be clarified that the formation of micro-vessels in the body of the matrix is an earlier stage of reconstruction, followed by osteogenesis [56,59,60]. At the same time, one of the important conditions for the rapid integration of building material is, in addition to degradation, high biocompatibility, the manifestation of its own angiogenic qualities, the ability to block polysaccharide and protein structures on the microbial cell surface, which provide transmembrane transport, and the ability to block pain conduction on somatic cells. These properties are known to be possessed by natural polysaccharide materials of cationic, anionic, or amphoteric structures. Modern approaches to solving the problem of vascularization also include the introduction of low molecular weight serum growth factors into the injury zone (for example, the serum growth factors in cattle “adgelon” used in this work) based on a biodegradable polymer framework, which plays the role not only of a depot of dislocation of growth factors but also of a program of their prolonged elution as a result of a certain rate of polymer biodegradation. Dosed elution of growth factors from a modern polysaccharide matrix forms new capillaries [61]. Uncovering the mechanisms of the initial stages of angiogenesis will solve the problem of critical early osteogenesis. Thus, early vascular endothelialization of the artificial matrix is a primary promising task and will mean the oriented sprouting of precursors or specialized cells, as well as signaling molecules of intercellular interaction, and, as a result, an active process of early osteogenesis [62]. Tight contact of the initially cell-free polysaccharide copolymer matrix with spongy and compact plates of the bone cavity with a certain filling of polymers with angiogenesis products triggers the process of matrix endothelization. Upon contact with the maternal vascular network, induced proliferation of the vascular endothelium and its translation into the sub-intimal zone occur, which culminates in an intensive neoplasm of micro-vessels. Under favorable conditions, this process captures the zone of the artificial matrix. The initial start of endothelialization ends with the rapid filling of the cell-free matrix with the vascular network. The osteoblastic and osteoclastic microenvironment of the vascular implant stimulates the rapid formation of cancellous and compact bone from the periphery to the center of the implant. It is very important that the artificially created vascular network does not regress after the complete degradation of the implant. The use of sodium alginate in the work was based on the results of modern research on the use of this polymer for tissue engineering [63,64,65]. However, weak mechanical strength and low activity of cell binding, especially in the hydrated state [66], low thermal and electrical conductivity, and antibacterial activity during the creation of the polymer scaffold required the inclusion of other polymers in this design. The inclusion of other polymers or inorganic compounds in the alginate significantly improves the mechanical and functional properties of the new design. This is especially important when solving the problem of eliminating a bone tissue defect of a critical size. It has been shown that the inclusion of hydroxyapatite in scaffolds based on sodium alginate increases the stability of the scaffolds and opens access to the cell surface [62], improves biocompatibility, changes the micro-relief of the matrix surface, and increases adhesion and migration of osteoblasts [29]. It has been shown [30] that scaffolds based on alginate and hydroxyapatite enhance the local elimination of bone defects since they do not cause inflammatory effects. The inclusion of various amounts of hydroxyapatite or alginate in the overall design reduces the rate of degradation in tissues.

It is known that the use of a chitosan membrane as a single polymer leads to the formation of new bone and cementum in single-wall intraosseous defects in large experimental animals [67]. Physical or chemical synthesis with other polymers significantly increases the mechanical strength and elasticity of the hydrogel [18]. The mutual penetration of individual polymers with the formation of hydrogel networks reinforces the structure, which is considered one of the best technologies for obtaining a composite with high mechanical properties [68]. Such combined meshes were developed using alginate and chitosan [19] or hyaluronic acid [20,21] for the purpose of bone tissue bioengineering. If a combination of chitosan with an alginate–hydroxyapatite framework is obtained [23,69,70], then the gel matrix is stabilized due to the formation of polyelectrolyte complexes. The complexes are formed as a result of a weak interaction of oppositely charged chemical groups of COO^−^ alginate and NH_3_^+^ of chitosan. In addition, NH_3_^+^ groups can interact with PO_4_^3−^ hydroxyapatite groups, linking the framework together and forming a more compact structure. In this case, porosity and degradation rate decrease, and mechanical stability increases [70]. If we are talking about the destruction of periodontal tissues in the experiment, then filling a bone defect with a complex of chitosan with a poly-anionic structure, for example, collagen [71], leads to inhibition of the apical migration of the epithelium and active formation of new bone and cementum. Such dynamics are due to the induction of the differentiation of mesenchymal cells into cement-forming osteoblasts. The introduction of additional natural polysaccharide polymers into the structure leads to obtaining not only excellent mechanical strength but, which is very important, to the active implementation of cellular functions such as adhesion, proliferation, and differentiation. For example, a composite scaffold consisting of a combination of chitosan, alginate, hydroxyapatite, and cellulose nanocrystals possesses such properties, which are especially valuable for bone tissue engineering. Attention should be paid to the use of sulfated polysaccharides such as heparin in the preparation of functional copolymers. Three sulfate groups in the heparin molecule allow for large rigid linear polymers to be converted into nanosized globules, which predetermines their high mobility in living animal tissues [6]. The high specific affinity of heparin for growth factors, for example, bFGF, is due to the presence of a mechanism of active electrostatic interaction between the negatively charged sulfate groups of heparin and the positively charged amino acid residues of the growth factor [29]. The high affinity of heparin for growth factors ensures high protein loading into the construct. The higher the growth factor loading, the higher the effect of angiogenesis. It has been established that bioactive growth factors must be slowly released from scaffolds to achieve long-term therapeutic effects [72]. Long-term dosed release into the medium, for example, of the main fibroblast growth factor (bFGF), significantly increases the angiogenic effect and capillary density in a short time [73,74]. However, this growth factor has a short half-life [75] and rapid diffusion from the injury site. Therefore, its practical application should be carried out in combination with a polysaccharide matrix. A copolymer alginate–chitosan structure containing additional anionic polysaccharides as a delivery system for growth factors can be a promising solution in the formation of a vascular bed not only in the bone cavity but also in the polymer matrix proper.

## Figures and Tables

**Table 1:** Histomorphometric criteria for the repair of a bone defect in the lower jaw of rats at an early follow-up (Me[25;75]).

	Area of Mandibular Defect		
Experiment Period	Experience CH–SA–HA ^1^	Control Healing under a Blood Clot ^1^	Defect Periphery ^1^ Control + Experience
Numerical density of inflammatory infiltrate (unit/0.043 mm^2^)			
3 day ^1^	56.1[46.4;67.6]	67.4[57.6;72.1] **	9.3[7.9;9.7] **
5 day ^1^	85.7[72.1;90.7]	93.7[86.1;99.4] **	14.6[13.4;15.7] **
7 day ^1^	88.1[74.6;91.9]	91.4[81.5;99.4] **	11.3[10.1;12.3] **
Volumetric density of bone tissue (BV (%))			
5 day ^1^	1.1[0.9;1.3]	1.1[0.8;1.5]	65.3[64.2;66.4] **
7 day ^1^	15.1[13.0;16.7]	16.2[14.9;17.9]	66.5[65.6;68.8] **
Trabeculae thickness (mm)			
5 day ^1^	0.04[0.03;0.05]	0.04[0.04;0.05]	0.15[0.15;0.16] **
7 day ^1^	0.06[0.05;0.07]	0.06[0.05;0.06]	0.15[0.15;0.16] **
Intertrabecular spaces (mm)			
5 day ^1^	0.61[0.60;0.63]	0.61[0.59;0.63]	0.21[0.21;0.23] **
7 day ^1^	0.43[0.42;0.45]	0.43[0.43;0.45]	0.20[0.19;0.21] **
Osteoblastic surface (%)			
5 day ^1^	70.5[66.4;74.8]	64.3[62.0;67.1] **	25.3[24.0;26.4] **
7 day ^1^	65.6[63.2;68.5]	59.2[57.9;60.2] **	21.8[20.2;23.5] **
Eroded bone surface (%)			
5 day ^1^	0.8[0.7;0.9]	1.0[.0.9;1.1] **	12.0[11.3;13.3] **
7 day ^1^	1.5[1.4;1.8]	1.9[1.6;2.1] **	7.9[7.7;8.0] **

** Differences are reliable when compared with the CH–SA–HA group (*p* < 0.01). ^1^ Significant differences in multiple comparisons ANOVA (*p* < 0.001).

**Table 2:** Histomorphometric criteria for the repair of a bone defect within 2–4 weeks after construct implantation (Me[25;75]).

	Area of Mandibular Defect		
Experiment Period	Experience CH–SA–HA ^1^	Control Healing under a Blood Clot ^1^	Defect Periphery ^1^ Control + Experience
Numerical density of inflammatory infiltrate (unit/0.043 mm^2^)			
2 weeks ^1^	26.8[24.2;28.5]	31.5[28.8;33.7] **	13.2[11.3;14.4] **
3 weeks ^1^	17.2[15.8;19.9]	17.5[15.7;22.6]	6.2[5.7;7.1] **
Volumetric density of bone tissue (BV %)			
2 weeks ^1^	30.0[28.8;31.0]	28.3[25.8;29.9] **	65.0[63.8;66.5] **
3 weeks ^1^	41.3[39.6;43.6]	38.6[36.1;40.2] **	67.8[67.0;69.3] **
4 weeks ^1^	62.9[60.7;66.8]	60.9[58.0;62.0] **	66.5[64.3;68.1] **
Trabeculae thickness (TT mm)			
2 weeks ^1^	0.12[0.11;0.13]	0.11[0.10;0.12] *	0.16[0.16;0.17] **
3 weeks ^1^	0.13[0.11;0.14]	0.12[0,12;0.13] *	0.15[0.14;0.16] **
4 weeks ^1^	0.16[0.15;0.17]	0.14[0.13;0.15] *	0.16[0.15;0.17] **
Inter-trabecular spaces (ITS mm)			
2 weeks ^1^	0.37[0.36;0.37]	0.39[0.38;0.40] **	0.22[0.22;0.24] **
3 weeks ^1^	0.29[0.28;0.30]	0.30[0.29;0.31] **	0.24[0.23;0.25] **
4 weeks ^1^	0.21[0.21;0.22]	0.22[0.21;0.22] **	0.21[0.20;0.22]
Osteoblastic surface (OS %)			
2 weeks ^1^	58.9[57.0;60.2]	49.9[49.5;50.4] **	13.1[12.4;13.7] **
3 weeks ^1^	50.8[49.0;53.1]	42.0[39.6;43.9] **	9.1[8.4;9.8] **
4 weeks ^1^	38.9[37.1;40.1]	36.0[34.7;37.0] **	7.2[6.3;7.8] **
Eroded bone surface (ES %)			
2 weeks ^1^	3.5[3.3;3.6]	3.9[3.7;3.9] **	6.1[5.5;6.9] **
3 weeks ^1^	5.9[5.6;6.1]	6.4[6.1;6.6] **	2.9[2.7;3.0] **
4 weeks ^1^	9.9[9.7;10.0]	10.7[10.5;11.1] **	1.3[1.2;1.4] **

* Differences are reliable with the CH–SA–HA group (*p* < 0.05). ** Differences are reliable when compared with the CH–SA–HA group (*p* < 0.01). ^1^ Significant differences in multiple comparisons ANOVA (*p* < 0.001).

**Table 3:** Histomorphometric criteria for the repair of a bone defect within 6–10 weeks after construct implantation (Me[25;75]).

	Area of Mandibular Defect		
Experiment Period	Experience CH–SA–HA ^1^	Control Healing under a Blood Clot ^1^	Defect Periphery ^1^ Control + Experience
Volumetric density of bone tissue (BV %)			
6 weeks ^1^	67.5[65.4;68.6]	62.6[60.0;64.2] **	68.7[67.2;69.8] *
8 weeks	67.1[63.2;69.1]	65.2[63.4;69.2]	67.0[65.4;68.5]
10 weeks	67.3[65.1;68.2]	68.3[66.6;69.2]	67.8[66.3;69.2]
Trabeculae thickness (TT mm)			
6 weeks	0.16[0.15;0.17]	0.16[0.15;0.16]	0.15[0.14;0.17]
8 weeks ^1^	0.16[0.15;0.16]	0.15[0.14;0.16] *	0.15[0.14;0.15]
10 weeks	0.16[0.16;0.17]	0.16[0.15;0.17]	0.16[0.16;0.17]
Inter-trabecular spaces (ITS mm)			
6 weeks ^1^	0.21[0.19;0.22]	0.21[0.20;0.22]	0.19[0.18;0.20] **
8 weeks ^1^	0.20[0.19;0.20]	0.21[0.19;0.21]	0.19[0.18;0.19] **
10 weeks ^1^	0.20[0.19;0.20]	0.22[0.21;0.23]	0.18[0.17;0.20] **
Osteoblastic surface (OS %)			
6 weeks ^1^	20.2[19.0;21.5]	16.4[14.9;18.0] **	4.0[2.8;4.7] **
8 weeks ^1^	8.7[8.1;9.9]	8.4[7.1;9.6]	1.5[1.3;1.8] *
10 weeks ^1^	3.6[2.8;4.0]	5.0[3.6;6.1] **	1.6[1.1;1.9] **
Eroded bone surface (ES %)			
6 weeks ^1^	4.8[4.6;4.9]	5.1[4.7;5.6]	0.8[0.7;0.9] **
8 weeks ^1^	2.6[2.5;2.8]	3.1[3.0;3.4] **	0.7[0.5;0.9] **
10 weeks ^1^	1.6[1.5;1.7]	1.3[1.2;1.4] **	0.9[0.8;0.9] **
Bone growth rate (% per day)			
1–28 days	2.24[2.17;2.39]	2.17[2.07;2.21] **	
6–14 days	28.9 [26.3;30.4]	27.2 [25.2;29.0] **	
15–28 days	3.23[2.85;3.36]	3.15[2.64;3.52]	
29–70 days	0.96[0.92;0.97]	0.97[0.95;0.99]	
1–70 days (total)	0.94[0.82;1.25]	0.96[0.85;1.15]	

* Differences are reliable with the CH–SA–HA group (*p* < 0.05). ** Differences are reliable when compared with the CH–SA–HA group (*p* < 0.01). ^1^ Significant differences in multiple comparisons ANOVA (*p* < 0.001).

## Data Availability

Not applicable.
